# Sirtuins as Potential Therapeutic Targets for Hepatitis B Virus Infection

**DOI:** 10.3389/fmed.2021.751516

**Published:** 2021-10-11

**Authors:** Fanyun Kong, Qi Li, Fulong Zhang, Xiaocui Li, Hongjuan You, Xiucheng Pan, Kuiyang Zheng, Renxian Tang

**Affiliations:** ^1^Jiangsu Key Laboratory of Immunity and Metabolism, Department of Pathogenic Biology and Immunology, Xuzhou Medical University, Xuzhou, China; ^2^Laboratory Department, The People's Hospital of Funing, Yancheng, China; ^3^Imaging Department, The Second Affiliated Hospital of Shandong First Medical University, Taian, China; ^4^Department of Infectious Diseases, The Affiliated Hospital of Xuzhou Medical University, Xuzhou, China; ^5^National Demonstration Center for Experimental Basic Medical Sciences Education, Xuzhou Medical University, Xuzhou, China

**Keywords:** HBV infection, HBx, sirtuins, therapy, molecular mechanisms

## Abstract

Sirtuins (SIRTs) are well-known histone deacetylases that are capable of modulating various cellular processes in numerous diseases, including the infection of hepatitis B virus (HBV), which is one of the primary pathogenic drivers of liver cirrhosis and hepatocellular carcinoma. Mounting evidence reveals that HBV can alter the expression levels of all SIRT proteins. In turn, all SIRTs regulate HBV replication *via* a cascade of molecular mechanisms. Furthermore, several studies suggest that targeting SIRTs using suitable drugs is a potential treatment strategy for HBV infection. Here, we discuss the molecular mechanisms associated with SIRT-mediated upregulation of viral propagation and the recent advances in SIRT-targeted therapy as potential therapeutic modalities against HBV infection.

## Introduction

Chronic infection with hepatitis B virus (HBV), which can cause cirrhosis and hepatocellular carcinoma (HCC), remains a serious global public health problem (1, 2). To date, two types of drugs-namely, interferon-α (IFN-α) and nucleos(t)ide analogs-have been approved for treating diseases caused by HBV (1, 3). Although clinical evidence has shown that these drugs have the capability to suppress viral replication and improve liver histology, current standard antiviral treatment strategies rarely cure HBV infection; moreover, drug resistance and disease recurrence after therapy cessation remain prevalent. Given the fact that the interaction between HBV and host cells determines the clinical outcomes of HBV infection, targeting cellular factors that contribute to viral replication is a clinically viable strategy to eliminate the virus. Sirtuins (SIRTs) are nicotinamide adenine dinucleotide (NAD)^+^-dependent histone deacetylases that regulate various biological processes, including stress responses, apoptosis, and metabolism (4, 5). In particular, emerging evidence shows that SIRTs play a significant role in chronic HBV infection (6–16). Here, we present a review on the role of HBV in the alteration of SIRTs, function of SIRTs in the modulation of viral replication, and therapeutic potential of SIRT-targeting strategies in the suppression of HBV infection.

## Effect of HBV on SIRTS

The SIRT protein was first discovered in *Saccharomyces cerevisiae* as a silent information regulator 2 (SIR2) that contributes to extension of life span under different types of stress (17–19). To date, a total of seven mammalian SIRT proteins have been identified. All seven SIRTs are present in all living organisms but with diverse intracellular localizations. SIRT1 and SIRT2 are present in the cell nucleus and cytoplasm; SIRT3, SIRT4, and SIRT5 are primarily localized in the mitochondria; and SIRT6 and SIRT7 are mainly present in the nucleus. Additionally, all SIRTs are members of class III histone deacetylases. In addition to their constitutive deacetylase activity, some SIRTs have alternative enzymatic activities, including ADP ribosyltransferase (SIRT4 and SIRT6), demalonylase (SIRT5), desuccinylase (SIRT5 and SIRT7), and demyristoylase (SIRT6) (18, 19). Given their diverse localizations and enzymatic activities, SIRTs are known to play a significant role in a wide range of biological functions. Owing to their ability to target various cellular substrates, SIRTs are crucial for numerous biological processes, including DNA repair, proliferation, mitochondrial energy homeostasis, and metabolism (17, 19). In particular, increasing evidence shows that SIRTs are implicated in the persistence and pathogenesis of viral infections (20) caused by human immunodeficiency virus, influenza A virus, herpes simplex virus 1, and human papillomavirus (18, 19). Considering the potential importance of SIRTs in viral infections, an improved understanding of the interaction between SIRTs and viruses may help researchers to develop alternative novel antiviral therapeutic agents.

To date, the effect of HBV on all proteins in the SIRT family has been extensively investigated by several groups. Notably, current evidence shows that the expression of all SIRT proteins can be modulated by HBV. In particular, expression levels of SIRT1 (6), SIRT2 (7), SIRT5 (8), and SIRT7 were found to be increased in HBV-infected cells (8, 9), whereas those of SIRT3, SIRT4, and SIRT6 were decreased in HBV-infected hepatocytes (10–12, 21). Of note, studies have also demonstrated that changes in the expression level of SIRT1 (14), SIRT2 (7), SIRT3 (11), SIRT4 (10), and SIRT7 are associated with a non-structural viral protein, HBx (9). Specifically, Wang et al. showed that, compared with those in the hepatoma cell line HepG2, the levels of SIRT1 mRNA and its protein were increased in HepG2.2.15 (HepG2 harboring HBV genome). Additionally, the HBV-mediated alteration in SIRT1 expression was further confirmed in HepAD38 cells, in which HBVs were continuously produced in the presence of tetracycline (14). Among the HBV-encoded proteins, only HBx has been found to increase SIRT1 expression at the mRNA and protein levels (14). The expression of HBx could also be upregulated by SIRT1 in the HBV-expressing hepatocytes. However, the cellular factors associated with the upregulation of SIRT1 mediated by viral proteins are still unknown.

Similar to the expression of SIRT1, SIRT2 and SIRT7 are increased in HBV-expressing hepatocytes (7, 9) although the mechanisms underlying the upregulation of SIRT2 and SIRT7 expression by the viral protein HBx is different. HBx-induced overexpression of SIRT2 mRNA and its proteins has been observed. Furthermore, HBx is capable of enhancing SIRT2 transcription by activating its promoters in the HBV-infected hepatoma cells (7). HBx has no effect on the expression of SIRT7 mRNA in hepatoma cells. However, the viral protein can interact and co-localize with SIRT7 (primarily in the nucleus), collectively enhancing SIRT7 stability by restraining its degradation that is regulated *via* the ubiquitin-proteasome pathway (9).

HBx suppresses the expression level of SIRT3 and SIRT4 in hepatoma cells (10, 11). A study showed that SIRT3 inhibition could increase the release of reactive oxygen species (ROS) induced by HBx (11). HBx-mediated SIRT4 suppression is related to an increase in cell cycle progression and the inhibition of apoptosis in hepatoma cells (10). Nevertheless, the detailed mechanisms of HBx-mediated SIRT3 and SIRT4 expression alterations are yet to be fully elucidated. Moreover, although HBV can modulate the expression levels of SIRT5 and SIRT6 (8, 21), the exact virus-encoded proteins that are responsible for the virus-mediated modification of SIRT5 and SIRT6 expression are still unknown.

## Function of SIRTS in HBV Replication

**Figure 1:**
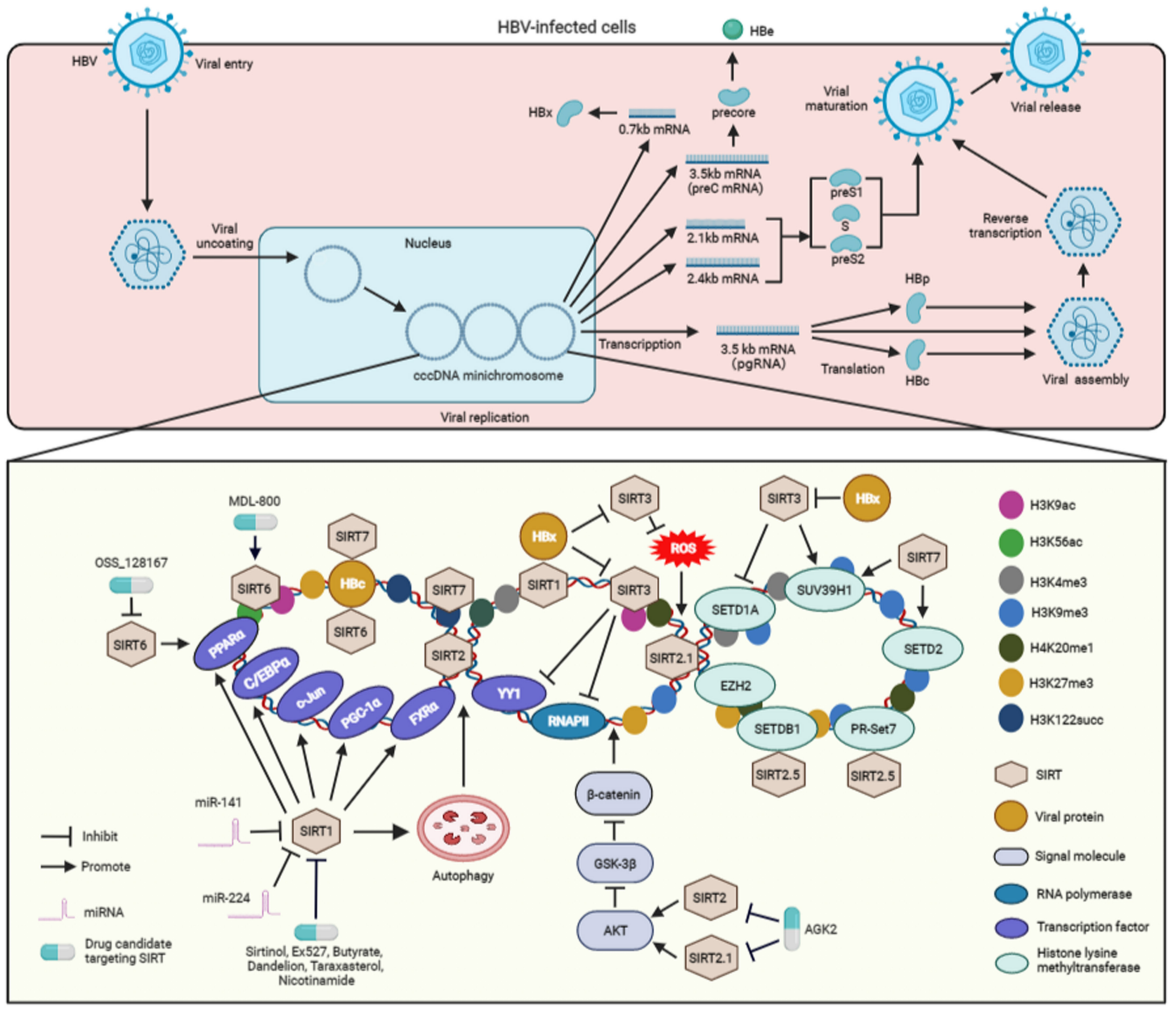
The molecular mechanisms associated with the regulation SIRT-mediated HBV replication and the SIRT-targeting antiviral agents to inhibit viral replication. After entering hepatocytes, HBV is uncoated and transferred into the nucleus and its genome is converted into cccDNA. Subsequently, the cccDNA forms a mini-chromosome and is transcribed into various viral mRNA, including pregenomic RNA (pgRNA) and preC mRNA, two envelope mRNAs, and X mRNA. The pgRNA serves as a translation template for HBc and viral polymerase proteins (HBp). The preC mRNA encodes pre-core protein, which is secreted as HBe antigen. X mRNA is translated to HBx protein. The two envelope mRNAs encode preS1, S, and preS2 domains to construct the various viral surface proteins. The pregenomic RNA is encapsulated into viral particles, and reverse-transcribed into viral DNA. The enveloped HBV particles are secreted from the HBV-expressing hepatocytes. During the cccDNA mini-chromosome-dependent HBV replication, SIRT1 interacts with HBx and then recruited to cccDNA. SIRT1 activates the transcription factors, inculding FXRα, PGC-1α, c-Jun, C/EBPα, and PPARα, which can promote the activation of HBV promoters, to facilitate viral replication. In addition, SIRT1 also enhances HBV replication via autophagy. However, miR-141 and miR-224 inhibit SIRT1 to block autophagy-mediated viral replication. SIRT2 and SIRT2.1 increase the replication of HBV via activating AKT, inhibiting GSK-3β, and then sensitizing the β-catenin signaling. SIRT2.5 recruits histone methyltransferases, including SETDB1, EZH2, SUV39H1, PR-Set7, and H3K27me3, H3K9me3, and H4K20me1 to cccDNA. The interactions of PR-Set7 and SETDB1 with SIRT2.5 are increased upon HBV replication. Upon recruitemnt to HBV mini-chromosome, SIRT3 can lead to an increase in histone lysine methyltransferases SUV39H1 but a decrease in SETD1A on viral cccDNA, which results in increased recruitment of H3K9me3 but decreased binding of H3K4me3 to HBV cccDNA. SIRT3-mediated viral cccDNA transcriptional repression is similar to the decreased recruitment of RNAPII and transcription factor YY1 on the viral cccDNA. HBx enhances cccDNA transcription by suppressing the expression of SIRT3 and inhibiting the recruitment of proteins to cccDNA. In addition, SIRT3 overexpression inhibits viral replication by reducing cellular ROS level. HBx can induce ROS production to promote viral replication, which is associated with the inhibition of SIRT3. SIRT6 activates the HBV promoters via upregulating PPARα expression. However, SIRT6 is able to repress HBV replication via interacting with HBc and downregulating H3K56 acetylation (H3K56ac) and H3K9 acetylation (H3K9ac) on the viral cccDNA mini-chromosome. SIRT7 binds to cccDNA by interacting with HBc and inducing the decrease of H3K122 succinylation (H3K122succ). Specifically, SIRT7 cooperates with SETD2 and SUV39H1 to induce the inhibition of viral transcription. The inhibitors, including sirtinol, Ex527, butyrate, dandelion, taraxasterol, and nicotinamide, can suppress viral replication via SIRT1-targeting. The inhibitor AGK2 suppresses HBV replication by blocking SIRT2 and SIRT2.1. OSS_128167, a SIRT6 inhibitor, can repress HBV replication. Whereas, MDL-800 can suppress viral replication by activating SIRT6. ROS, reactive oxygen species; RNAPII, RNA polymerase II; H3K9me3, trimethyl-H3K9; H4K20me1, monomethyl-H4K20; H3K27me3, trimethyl-H3K27; H3K4me3, trimethyl-H3K4; H3K9ac, H3K9 acetylation; H3K56ac, H3K56 acetylation; H3K122succ, succinylation of histone H3K122; SETDB1, SET domain bifurcated 1; EZH2, enhancer of zeste homolog 2; SETD1A, SET domain containing 1A; SUV39H1, suppressor of variegation 3–9 homolog 1; cccDNA, covalently closed circular DNA.

The replication cycle of the small enveloped double-stranded circular DNA of HBV has been fully deciphered (Figure 1) (3, 22). The covalently closed circular DNA (cccDNA) mini-chromosome of HBV acts as the central replication and transcription template and thus plays a central role in its replication (3). Although all viral transcripts are derived from HBV cccDNA, elements such as viral proteins, transcriptional regulators, and epigenetic modulators are known to regulate the transcription of cccDNA. Specifically, the HBV cccDNA mini-chromosome is stably maintained in host hepatocytes to facilitate the persistence of HBV infection. In particular, current studies have shown that not only can SIRT1 interact with HBx (13) but also can both SIRT1 and HBx be recruited to the viral cccDNA, resulting in a more robust production of cccDNA, pregenomic RNA, as well as surface antigen. To date, various research groups have investigated the effect of SIRT1 on HBV replication. Li et al. showed that SIRT1 facilitates HBV replication by activating all viral promoters located in the HBV cccDNA, including the core, X, preS1, and preS2 promoters (15). Moreover, previous studies have indicated that SIRT1 can sensitize the FXRα, PGC-1α, and c-Jun to enhance viral transcription *via* the activation of HBV core promoter (6, 16). In addition, C/EBPα and PPARα, which contribute to the activation of the core, X, preS1, and preS2 promoters (23), participate in the SIRT1-mediated HBV replication (15, 24) (Figure 1).

Autophagy is an important non-selective self-degradation physiological process, by which cell constituents are sequestered in autophagosomes that consecutively fuse with lysosomes to facilitate substrate degradation (25). A growing body of evidence shows that SIRT1 induces autophagy by directly deacetylating autophagy-related molecule markers, including ATG5, ATG7, and ATG8 (26). Of note, studies have demonstrated that autophagic response is crucial for HBV replication (27), and particularly, Yang and Gao reported that SIRT1 facilitates HBV replication through viral-induced autophagy (28, 29). However, miR-141 and miR-224 may play a role of suppressing autophagy-induced HBV replication by targeting SIRT1 (Figure 1). Yamai et al. showed that SIRT1 is capable of promoting autophagy-induced HBV replication, the role of SIRT in viral replication is independent of autophagy (30). Therefore, further investigation is required to clarify the exact role of autophagy in SIRT1-dependent HBV replication. In addition to SIRT1, studies have demonstrated that other SIRTs, including SIRT2, SIRT3, and SIRT 6, can also induce autophagy *via* alternative molecular mechanisms (5, 31). However, it remains ambiguous whether these SIRTs can regulate HBV replication *via* autophagy, and therefore, warranting further studies.

A study from Piracha et al. indicates that HBV can increase the expression level of SIRT2 as well as its alternatively spliced transcripts SIRT2.1 and SIRT2.5 (32). It has been demonstrated that the activation of AKT, a core molecule in the PI3-K pathway, is involved in the replication of HBV (33). As a deacetylase, SIRT2 interacts with AKT to enhance its activation *via* deacetylation (34). In particular, Piracha et al. showed that, *via* their interaction with AKT, SIRT2 and SIRT2.1 induce AKT activation to downregulate GSK-3β, which in turn upregulates β-catenin expression to increase the transcriptional activity of HBV (35). Unlike SIRT2.1, SIRT2.5 could only slightly activate the AKT/GSK-3β/β-catenin signaling pathway. However, SIRT2.5 plays an exact opposite role of SIRT2.1, which is to reduce the expression level of HBV cccDNA and mRNAs. Studies have further revealed that, unlike SIRT2.1, SIRT2.5 is primarily recruited to HBV cccDNA (32). The recruitment of histone lysine methyltransferases, such as enhancer of zeste homolog 2 (EZH2), suppressor of variegation 3–9 homolog 1 (SUV39H1), SET domain bifurcated 1 (SETDB1), and PR-Set7, and the methylation of distinct histones, including trimethyl-H3K9 (H3K9me3), monomethyl-H4K20 (H4K20me1), and trimethyl-H3K27 (H3K27me3), are also increased in the cccDNA of SIRT2.5-overexpressing HBV (32). Among these histone lysine methyltransferases, SIRT2.5 could interact with PR-Set7 and SETDB1 on cccDNA, and these interactions are increased upon the replication of HBV (Figure 1). As mentioned earlier, Cheng et al. demonstrated that SIRT2 expression could be upregulated by HBx to promote HBV replication (7). However, Piracha et al. showed that SIRT2-mediated upregulation of HBV replication are independent of HBx (32, 35). Taken together, the findings on the mediating effect of SIRT2 on HBV replication obtained by different researcher groups remain inconclusive. Therefore, further investigation is vital to examine the exact role of SIRT2 and its alternatively spliced transcripts in HBx-mediated HBV replication.

Unlike SIRT1 and SIRT2, SIRT3 exerts an inhibitory effect on HBV replication by targeting the cccDNA *via* epigenetic regulation. Specifically, SIRT3 can induce a decrease in H3K9 acetylation (H3K9ac) on viral cccDNA, leading to an increase in histone lysine methyltransferase SUV39H1 and a decline in the SET domain containing 1A (SETD1A) on cccDNA. The recruitment of SIRT3 to viral cccDNA also results in an increase in H3K9me3 and a decrease in trimethyl-H3K4 (H3K4me3) on HBV cccDNA. Furthermore, the transcriptional repression of HBV cccDNA mediated by SIRT3 is also related to a decrease in RNA polymerase II (RNAPII) and the transcription factor YY1 on cccDNA (Figure 1). However, HBx can relieve cccDNA transcriptional repression by restricting the expression of SIRT3 and blocking the recruitment of the proteins to cccDNA (12). As a major mitochondrial deacetylase, SIRT3 plays a crucial role in the regulation of ROS by targeting proteins involved in mitochondrial functions and antioxidant defenses (36). Ren et al. reported that SIRT3 can also inhibit HBV replication by decreasing ROS levels in the HBV-expressing hepatocytes. In contrast, HBx can induce ROS to promote viral replication, a process similar to viral protein-mediated SIRT3 inhibition (11).

Findings from two separate studies by Deng et al. and Jiang et al. showed that SIRT6 can promote HBV replication and activate the HBV core promoter *via* upregulating the expression of transcription factor PPARα (13, 37). However, the latest evidence from Yuan et al. indicated that SIRT6 is a restricting factor of HBV. SIRT6 can interact with HBV core protein (HBc) and suppress viral replication through its deacetylase activity by inhibiting H3K56 acetylation (H3K56ac) and H3K9 acetylation (H3K9ac) on the HBV cccDNA mini-chromosome (21) (Figure 1). The reasons for the contrasting effect of SIRT6 on HBV replication by different researchers are unclear. Therefore, further investigations are warranted to examine the exact role of SIRT6 in HBV replication.

Although SIRT7 is involved in the modulation of HBV replication, results on the effect of SIRT7 on HBV replication reported by different groups remain inconclusive. Deng et al. showed that SIRT7 contributes to HBV replication (13), but the molecular mechanisms that contribute to the replication of HBV remains ambiguous. Yu et al. reported that SIRT7 can bind to cccDNA by interacting with the HBc protein. Due to its desuccinylase activity, SIRT7 induces a decrease in histone H3K122 succinylation (H3K122succ) on the cccDNA (Figure 1). Furthermore, SIRT7 can act cooperatively with histone lysine methyltransferase SUV39H1 and SETD2 to modulate the chromatin structure of cccDNA and facilitate the inhibition of HBV transcription (38). Similar to SIRT6, further studies are vital to investigate the exact role of STIR7 in HBV replication. Although SIRT4 and SIRT5 can enhance HBV replication (13), the exact molecular mechanisms mediated by these two SIRTs are still not fully clarified.

## SIRTS as Potential Therapeutic Targets in HBV Infection

Owing to the growing evidence on the importance of SIRTs in HBV infection, targeting SIRTs using antiviral agents is an appropriate strategy to attenuate the replication of HBV (Figure 1). For example, inhibition of SIRT1 with sirtinol and Ex527 has a significant antagonistic effect on the replication of HBV (13). Emerging data indicate that butyrate can inhibit HBV replication by targeting SIRT1 (39). In addition, dandelion and taraxasterol, which have the role of targeting SIRT1, also exert a significant inhibitory effect on HBV replication (40). Additionally, nicotinamide, another SIRT1 inhibitor, could suppress HBV replication *in vitro* and *in vivo* (15).

As mentioned above, SIRT2 and SIRT2.1 have been demonstrated to accelerate viral replication (32, 35), and AGK2 could suppress HBV replication by targeting SIRT2 and SIRT2.1 *in vitro* and *in vivo* (35, 41). Jiang et al. and Yuan et al. reported contradicting effect of SIRT6 on the replication of HBV (21, 37). The inhibitor or activator of SIRT6 exhibited an inhibitory effect on HBV replication in these two studies. Jiang et al. demonstrated that the selective inhibitor OSS_128167 could target SIRT6 to restrict viral replication in both HepG2.2.15 cells and HBV-transgenic mice (37). However, data from Yuan et al. indicated that a specific activator of SIRT6, MDL-800, can suppress the replication of the virus *in vitro* and *in vivo* (21) (Figure 1). Therefore, further study is vital to examine the exact effect of SIRT6 inhibitors or activators on HBV replication.

## Conclusion and Future Perspectives

Currently, the standard treatment regimens for HBV are mainly limited to IFN-α and nucleos(t)ide analogs. IFN-α treatment strategy is primarily employed to elicit cytokine-induced antiviral immune response *via* the expression and antiviral activity of IFN-stimulated genes. Nucleos(t)ide analogs inhibit viral replication *via* their accumulation on the viral genome and thereby disrupt the transcription of viral polymerase (1, 42). However, these two drugs cannot cure the HBV-induced diseases and often result in serious side effects, drug resistance, and disease recurrence. Therefore, novel molecular targets for HBV treatment are urgently needed. Based on this review, we infer that HBV can alter the expression of SIRTs and that SIRTs are capable of promoting viral replication *via* multiple pathways. First, SIRTs regulate HBV replication by controlling the activity of viral promoters that rely on various transcription factors, including FXRα, PGC-1α, and c-Jun. Second, SIRTs can modulate many biological processes, including the induction of autophagy, increase in ROS production, and activation of signaling pathways to regulate HBV replication. Third, SIRTs can also modify the chromatin structure of HBV cccDNA *via* epigenetic regulation based on histone methylation, acetylation, and succinylation to modulate viral replication. Besides these, the utilization of different inhibitors or activators associated with SIRTs can effectively inhibit HBV replication *in vitro* and *in vivo*. Therefore, targeting SIRTs, as a means to disrupt the biological processes that benefit viral replication *via* suppressing the activation of HBV promoters and altering the chromatin structure of viral cccDNA, is a promising treatment strategy against HBV infection.

In the search for potential novel SIRTs antagonists, many significant breakthroughs have been attained in recent years (43, 44). Importantly, several preclinical and clinical trials have been conducted to assess the effect of SIRT-targeting drug candidates for the treatment of various diseases, including diabetes, obesity, and cancer (43, 45). Nevertheless, the use of SIRT-targeting drug candidates may cause other side effects attributed by the interacting effect between the drugs on other biological processes in which SIRTs are involved. Although some SIRT-targeting agents, including EX-527 and resveratrol, are reported to be safe and well-tolerated in various clinical trials (45–47), mild adverse effects, including dizziness, headache, and epididymitis, have been frequently reported in some participants administered with resveratrol (45). Therefore, to better assess whether targeting SIRTs is a novel promising treatment strategy against HBV-induced diseases, large scale clinical trials in the near future are vital. In addition, not only the clinical efficacy but also the safety and tolerability of the SIRT-targeting antagonist candidates in patients with HBV infection should be verified.

HBx is a non-structural viral protein that plays a vital role in the replication of HBV (22). Our review provides clear evidence that the HBV-mediated overexpression of SIRTs to regulate viral replication is primarily HBx-dependent. However, the exact molecular mechanisms underlying the HBx-induced alteration of SIRT expression are still not fully elucidated. To better understand the exact effect of SIRTs on HBV replication regulated by the viral proteins, further investigation on the interactions between HBx and SIRTs is needed. In this review, our analysis of current data was primarily focused on the effect of SIRTs on HBV replication; the role of SIRTs in the development of cirrhosis and HBV-induced HCC remains poorly understood. Therefore, it is crucial to further examine the functions and molecular mechanisms of SIRTs in regulating the development of HBV-induced diseases.

## Author Contributions

All authors listed have made a substantial, direct and intellectual contribution to the work, and approved it for publication.

## Funding

The study was supported by the Priority Academic Program Development of Jiangsu Higher Education Institutions (PAPD). The figures presented in the study were created using BioRender (https://Biorender.com/).

## Conflict of Interest

The authors declare that the research was conducted in the absence of any commercial or financial relationships that could be construed as a potential conflict of interest.

## Publisher's Note

All claims expressed in this article are solely those of the authors and do not necessarily represent those of their affiliated organizations, or those of the publisher, the editors and the reviewers. Any product that may be evaluated in this article, or claim that may be made by its manufacturer, is not guaranteed or endorsed by the publisher.
